# Camouflaged angiogenic BMP-2 functions exposed by pico-paracrine biohybrids

**DOI:** 10.3389/fbioe.2023.1226649

**Published:** 2023-09-07

**Authors:** Herbert P. Jennissen

**Affiliations:** Institute of Physiological Chemistry, Faculty of Medicine, University of Duisburg-Essen, Essen, Germany

**Keywords:** angioinduction, osteoinduction, pico-technology, constitutive receptor activity, allosteric inverse agonist, orthosteric agonist, inverse concentration dependence, proangiogenic and antiangiogenic factors

## Abstract

The constant release of human bone morphogenetic protein 2 (rhBMP-2) in the picomolar range (Pico-Stat) from PDLLA-biohybrids led to the detection of intrinsic novel pro- and anti-angiogenic functions of this cytokine. As integrant part in this perspective of previous work, first evidence for the binding of rhBMP-2, as an *inverse agonist*, to allosteric angiogenic receptors in cocultures of human endothelial cells is reported.

## 1 Introduction

BMP-2 initiates osteogenesis requiring angiogenesis, with the ingrowth of mesenchymal stem cells and osteoblasts. The global market size (MRI, 2023) of the recombinant form (rhBMP-2) [INFUSE^®^ Bone Graft (Food and Drug Administration, 2004)] reached USD 498.1 million in 2021 (MRI, 2023). This protein, rhBMP-2_CHO_, is produced by a genetically engineered Chinese hamster ovary cell line, as a disulfide-linked heterodimeric protein mixture of two different polypeptide chains of 114 and 131 amino acids. Each chain is glycosylated at one site with high-mannose-type glycans (Food and Drug Administration, 2004; Committee for Medicinal Products for Human use, 2014; Kenley et al., 1993). As applied, BMP-2_CHO_ comprises a microheterogenous mixture of three different BMP-2 isoforms and up to 15 additional different glycoforms (Kenley et al., 1993).

**FIGURE 1:**
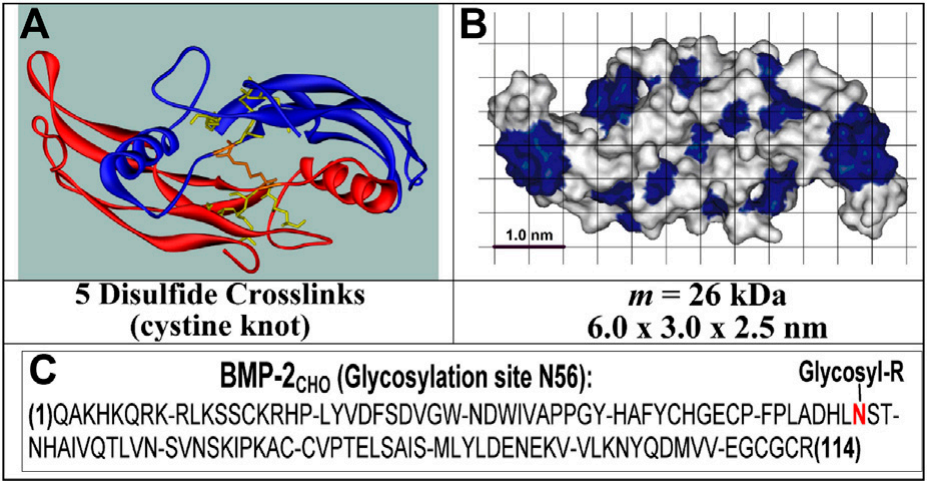
Structures of rhBMP-2_COL_. **(A)** Ribbon structure of 2 identical polypeptides; **(B)** solvent-accessible surface structure with blue hydrophobic and white hydrophilic patches; **(C)** Primary structure with N56 glycosylation site [see (Laub et al., 2001; Chatzinikolaidou et al., 2002)].

In contrast, the non-glycosylated rhBMP-2_COL_ second species (Schlüter et al., 2009) from *E. coli* (Figure 1) is homodimeric and displays no microheterogeneity (Jennissen et al., 1999; Laub et al., 2007). The molecular masses of the commercial glycosylated (rhBMP-2_CHO_) and the non-glycosylated rhBMP-2 species (rhBMP-2_COL_) correspond to 33–36 kDa for the former and 26 kDa for the latter, respectively (Laub et al., 2007). In humans, 12 mg of rhBMP-2_CHO_ must be applied for osteoinduction in a concentration of 1.5 mg/mL (∼ 44 µM) in an absorbable collagen sponge (ACS). After intravenous injection, rhBMP-2 is eliminated with a half-life of t_1/2_ = 6.7 min in nonhuman primates (Food and Drug Administration, 2004). Physiological serum levels of BMP-2 in healthy human controls are reported to be 17.1 ± 0.6 pg/mL (= 4.8 × 10^−13^ M) (Kercheva et al., 2020).

In receptor-binding studies, the binding function (θ) and the state function (r) are distinguished (Laub et al., 2007). The binding function (θ) of rhBMP-2 is a direct measure of receptor occupation by the ligand with K^θ^_D_ in the range of ∼0.45 nM (Mayer et al., 1996). The state function (r) (Wiemann et al., 2001; Laub et al., 2007) of rhBMP-2 correlates with the receptor activity state by monitoring downstream products (e.g., alkaline phosphatase) in activity tests in MC3T3-E1 cell cultures, with both species of rhBMP-2_CHO_ and rhBMP-2_COL_ having indistinguishable biological activities of nanomolar affinities (K^r′^_D_ ∼ 2–10 nM (Laub et al., 2007)). BMP-2 also specifically binds to its prodomain for ECM targeting, presumably with a similar affinity constant (K^′^_D_ ∼ 4–8 nM) as BMP-7 (Spanou et al., 2023). RhBMP-2 is known to indirectly initiate angiogenesis in the nanomolar range (0.38–3.8 nM) (Deckers et al., 2002) by activating paracrine VEGF-mediated osteoblast‐endothelial cell cross‐talk (Kulikauskas and Bautch, 2022). The combination of biomolecular materials, such as rhBMP-2, with the other biomaterial classes such as metals, polymers, or ceramics, forms a biohybrid material [see (Jennissen, 2019)]. In protein immobilization, we distinguish adsorbates (adsorption), covalates (covalent binding), and inclusates (encapsulated biohybrids) (Jennissen, 2019). A biohybrid is classed as bioactive if it has been “designed to induce a specific biological activity” (Williams et al., 1987). A paracrine biohybrid is, e.g., a growth factor releasing biomaterial. Recently, we reported the first evidence of a direct influence of rhBMP-2_COL_ on angiogenesis in co-cultures at picomolar concentrations (Dohle et al., 2021). Applications of rhBMP-2 in solute and PDLLA solid-phase forms are described.

## 2 Methods

RhBMP-2_COL_ from *E. coli* (Morphoplant GmbH Bochum, D) and rhBMP-2_CHO_ from CHO-cells (InductOs^®^, Medtronic BioPharma B.V., Heerlen, NL) were employed (Laub et al., 2007). The dose–response derived biological equivalent activity equals K'_D_ ∼ 5–15 nM for both species of rhBMP-2 (Laub et al., 2007). The for many weeks stable PDLLA nanofiber fleece preparation is described in (Sowislok and Jennissen, 2022). Labeled ^125^I-rhBMP-2 (Dohle et al., 2021) was for the analytical preparation of adsorbate PDLLA biohybrids. Non-isotope biohybrids were made in parallel for the other experiments. In a 24-well cell culture plate, the single well volume corresponds to 0.5–1.0 mL. Human outgrowth endothelial cells (OECs) and human primary osteoblasts (pOBs) were prepared as described (Dohle et al., 2021). Cocultures consisted of endothelial cells (130.000/well) and primary osteoblasts (20.000/well). Relative gene expressions (RQ) of mRNA for various proteins are described in (Dohle et al., 2021). In dose-response experiments*,* rhBMP-2_COL_ was added in defined concentrations to the OEC/pOB co-culture system and harvested after 7 days of incubation for fluorescence microcopy and quantitative Nikon NIS image processing as described (Dohle et al., 2021). The pixel values were converted to length according to 1 pixel = 0.46 µm. Cell viability was tested by the LDH cytotoxicity assay CyQUANT™ as described (Dohle et al., 2021). In desorption experiments, 1 cm^2^ (1 mg) fleeces with an rhBMP-2_COL_ loads of ∼ 6.98 mg/g PDLLA were placed in 1.5 mL flow-through chambers (Sänger et al., 2014). They were perfused with sterile phosphate-buffered saline (PBS) pH 7.4 at a flow rate of 10 mL/h in a 16-channel Watson-Marlow peristaltic pump for 22 days at ca. 22°C-26°C. Statistical calculations were done with the PC program GraphPad Prism 4/5 (La Jolla, CA). Prism software fails in exponential decays with r^2^-values of <0.2 when decays parallel the abscissa at t_1/2_ > 100–200 days (slope ∼ zero). Kinetic and thermodynamic constants under non-standard conditions are termed “apparent” (K′, k′). Equilibrium dissociation constants (K_D_, K_0.5_) represent affinity constants (Landry et al., 2015).

## 3 Results

### 3.1 Pico-paracrine biohybrids

In preparing solid-state paracrine adsorbate biohybrids, rhBMP-2_COL_ was adsorbed to the surface of a non-woven fleece composed of PDLLA nanofibers (∅ = ∼ 100 nm), prepared by electrospinning (Dohle et al., 2021; Sowislok and Jennissen, 2022) (see Table 1).

**TABLE 1:** Preparation of rhBMP-2-PDLLA-adsorbate biohybrids*.

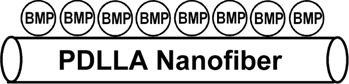		
BMP-2 [µg/mL] concentration	Adsorbed amount [mg/g]	
	rhBMP-2_COL_	rhBMP-2_CHO_
10 (low)	0.33 ± 0.06	0.58 ± 0.10
30 (high)	2.61 ± 0.81	3.40 ± 0.61

* The adsorbed load (high and low) to the nanofiber fleece from the adsorption solution (10 and 30 μg/mL) was determined at room temperature after 10–20 h of adsorption by tracer detection with^125^1-rhBMP-2 (Dohle et al., 2021). Mean ± SD, *n* = 3.

**FIGURE 2:**
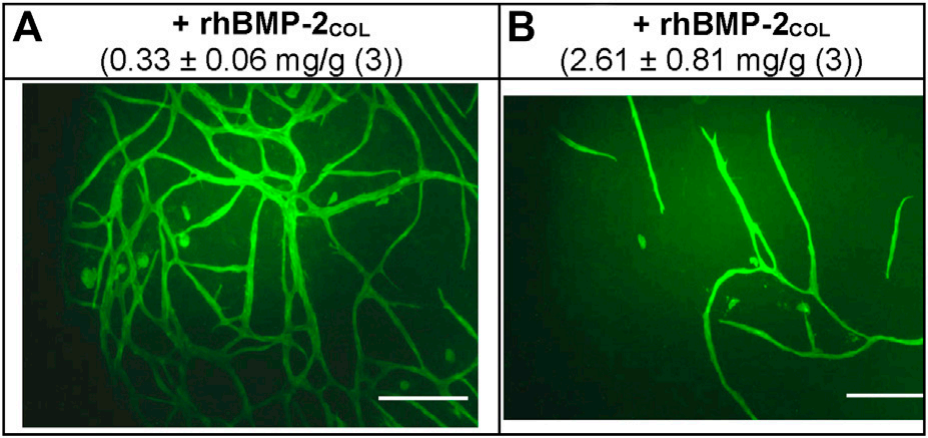
Angioinduction by paracrine rhBMP-2 PDLLA-nanofiber adsorbate biohybrids. **(A)** at low and **(B)** at high adsorbate load after 14 days in *OEC/pOB* co-culture system (magn. x10). Endothelial cells were immunofluorescently stained for load and CD31. For load (1.0 mg/g = 1.8 μg/cm^2^), and intrinsic activity of fleeces, see Tables 1, 3, scale: 300 μm; [from (Dohle et al., 2021)].

Decisive is the 22-day rhBMP-2_COL_ adsorbate release kinetics from biohybrids loaded with ∼8.9 mg/g Table 2. The two-phase exponential decay function displays a half-life of 3–4 h for the burst phase (K′_D_ = 8.2 nM) and a half-life of 208 days for sustained high-affinity release (K′_D_ = k_−2_/k_+1_ = 0.59 × 10^−12^ M; = pico paracrine biohybrid). Such rhBMP-2_COL_ adsorbate PDLLA biohybrids (Table 1) were incubated in OEC/pOB co-cultures for 14 days and led to large numbers of capillary–like microvessels with no VEGF-A gene activation (Dohle et al., 2021) (Figure 2A; 0.33 mg/g). Fewer microvessels, but more than in controls, form at an 8-fold higher rhBMP-2_COL_ adsorbate load (Figure 2B).

**TABLE 2:** ^125^I-rhBMP-2_COL_ release kinetics from biohybrids (Table 1).

Initial 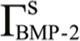	Burst			Sustained release		
	k′_-1_	t_1/2_	K′_D_	k′_-2_	t_1/2_	K′_D_
mg/g	10^−4^ [s^−1^]	[d]	nM	10^−8^ [s^−1^]	[d]	pM
8.97	5.34	0.015	8.2	3.86	208	0.59
±	±			±		
1.53	0.16			2.91		

* Data are derived from desorption kinetics of a 22-day duration [for details see (Sänger et al., 2014)] $\bar{x}\pm$ SEM; for k_+1_ = 6.5 ± 0.4 × 10^4^ M^−1^ s^−1^ see (Dohle et al., 2020).

In contrast to the pro-angiogenic activities of rhBMP-2_COL_, the glycosylated species rhBMP-2_CHO_ was anti-angiogenic, fully inhibiting control angiogenesis (Dohle et al., 2021) and forming instead a cobblestone layer of endothelial cells [not shown, see (Dohle et al., 2021)].

These results were confirmed in Table 3. At a low rhBMP-2_COL_, adsorbate loads of 0.33 mg/g and a total length of microvessels of 23.9 ± 5.1 mm formed without an increased expression of VEGF-A (Dohle et al., 2021). At high adsorbate loads of 2.6 mg/g, the total length decreased 5-fold to 4.7 ± 0.44 mm, and in controls lacking adsorbate down 10-fold to intrinsic 2.4 ± 0.046 mm. The knots in the microvessel mesh paralleled the length changes. At low concentrations of rhBMP-2_COL_, they totaled a high of 51.9 ± 11.5 knots, decreasing 4.3-fold to 12.2 ± 0.6 at higher concentrations and 12-fold less in controls of 4.3 ± 1.1 knots. These changes were statistically significant (Table 3).

**TABLE 3:** Length and knot data of capillary-like structures induced by rhBMP-2_COL_ adsorbate biohybrids after 14 days in co-culture*.

rhBMP-2_COL_ Biohybrid	Adsorbed amount of rhBMP-2_COL_		
	*Ø* mg/g Controls	0.33 mg/g	2.6 mg/g
(After 14 days)	(intrinsic activity)	(pro-angiogenic activity)	
Length Analysis (mm)	2.4 ± 0.046	23.9 ± 5.1	4.7 ± 0.44
Knot Analysis (dimensionless)	4.3 ± 1.1	51.9 ± 11.5	12.2 ± 0.6

* mean ± SD, *n* = 4; in t-tests all values of a series differ significantly *p* < 0.001 from one-another [see (Dohle et al., 2021) Table 1 and Figure 2].

### 3.2 Solution studies

In the following, concentration-response experiments in solution are shown. Instead of correlating with rising concentrations, the pro-angiogenic microvessel response increased as a function of factor-10 graded dilutions of rhBMP-2_COL_ (Table 4). Microvessel formation thus followed an inverse concentration gradient, exposing a hitherto unseen angiogenic activity by rhBMP-2_COL_ in the picomolar range, confirming results in Figure 2 and Table 3. Anti-angiogenic concentrations of rhBMP-2_COL_ >10 pM fully abolished pro-angiogenic responses.

**TABLE 4:** Dose-response angioinduction in OEC/pOB co-cultures after 7 days by decreasing concentrations of rhBMP-2_COL_ in solution*.

[rhBMP-2_COL_]	Microvessel total length		
	Gross, pixel	Net, pixel	Net, mm
Control, 0	13,070 ± 1,456	-	-
1 × 10^−7^ M	8 ± 0.6	0	0
1 × 10^−8^ M	12 ± 3	0	0
1 × 10^−9^ M	22 ± 1	0	0
1 × 10^−10^ M	1,217 ± 436	0	0
1 × 10^−11^ M	16,360 ± 377	3,290 ± 75	1.5 ± 0.015
1 × 10^−12^ M	42,103 ± 4,276	29,034 ± 2,961	13.4 ± 1.4
1 × 10^−13^ M	46,245 ± 3,978	33,175 ± 2,853	15.3 ± 1.3

* Microvessels (capillary-like structures) were determined by quantitative histologic staining (CD31; 1 pixel ∼0.46 µm). For further details see Figure 2, (mean ± SD, *n* = 3). [data from (Dohle et al., 2021)].

**FIGURE 3:**
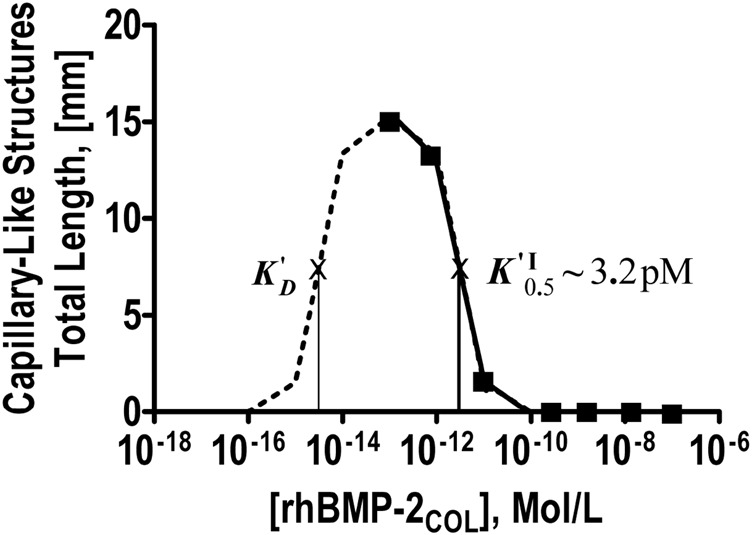
Dose-response-curve of angioinduction by rhBMP-2_COL_ after 7 days in OEC/pOB co-cultures. Inverse concentration dependence, half-maximal autoinhibition constant K^I^_0.5_, and a hypothetical K′_D_ on a hypothetical dotted association line are shown (see Table 4).

The data of Table 4 are plotted in Figure 3 down to concentrations of 10^−16^ M for a classical receptor type. Inhibition increases with the concentration of rhBMP-2_COL_. The apparent half-saturation constant is not a classical affinity constant but an “autoinhibition” constant (K′^I^_0.5_) with a value of 3.2 pM. The hypothetical (see dotted line) dissociation constant (K′_D_) for the presumed association of rhBMP-2_COL_ to endothelial cell receptors lies in the subpicomolar range ∼10^−14^ M. The anti-angiogenic regulation by rhBMP-2_COL_ prevents too many blood vessels from being formed as a “high concentration cut-off regulation”, in which an apparent agonist turns into an antagonist at high concentrations.

**FIGURE 4:**
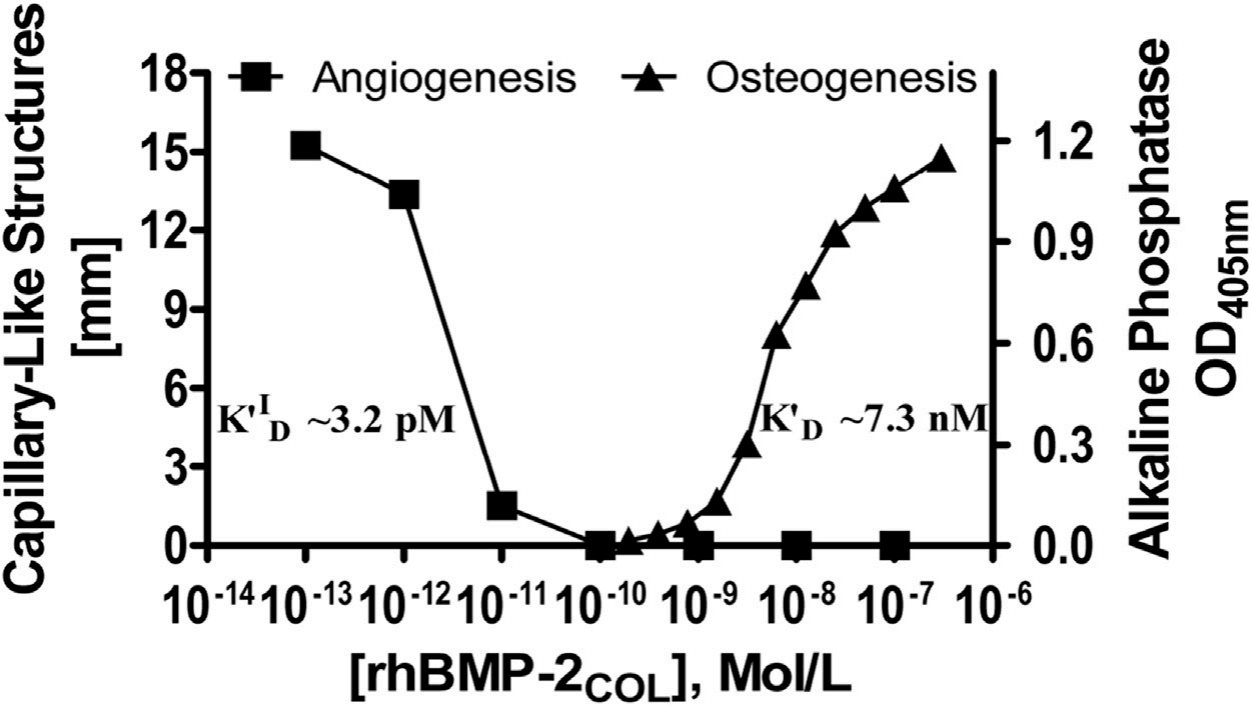
Dose-response curves of rhBMP-2_COL_ for the induction of angiogenic (inverse agonist) and osteogenic activities. Cocultures of OEC/pOB cells (Dohle et al., 2021) and mono-cultures of MC3T3-E1 cells (Laub et al., 2007) were employed respectively with corresponding affinity constants K′^I^_D_ (see also legends to Table 4 and Figure 3).

Figure 4 shows a compound extended dose-response plot comprising the data of Table 4 on inverse related angioinduction (Dohle et al., 2021) together with data on direct related osteoinduction [derived from activity measurements with MC3T3-E1 cells in culture (Laub et al., 2007)] and their respective affinity constants. The inverse angiogenic branch saturates at ca. 15 mm and the direct osteogenic branch at 1.2 OD units. The apparent half-saturation autoinhibition constant (K^I^_0.5_) can now be termed as an inverse-related affinity constant (K^′^_D_^I-R^) for angiogenesis with a value of 3.2 pM which contrasts with the direct-related affinity constant (K^′^_D_) for osteoinduction at 7.3 nM. Since the constants of the two activities are separated by three and more orders of magnitude, identical receptors, shared, cross-reactive receptors and homologous desensitization (Popovic and Wilson, 2010) of receptors are improbable, strongly indicating an angiogenic receptor with *constitutive activity* (Berg and Clarke, 2018) (see saturation in Figure 4) and rhBMP-2_COL_ as a *full inverse agonist* in a typical dose response curve in Figure 4.

Control of local picomolar concentrations of rhBMP-2_COL_ in solute form (Table 4) are short-term and difficult, but via solid-state technology, e.g., as rhBMP-2_COL_-PDLLA biohybrids (Figure 2; Table 3), are simple and long-term by sustained release (Table 2), comparable to a pH-Stat. In this study, not the pH but the growth factor concentration in the pico- or subpicomolar range is maintained as constant and guaranteed by high-affinity dissociation constants K′_D_ (= Pico-Stat) (Jennissen, 2023).

### 3.3 rhBMP-2 an angiogenesis inhibitor

As shown, the microvessel control values in the absence of rhBMP-2 (experiments of Tables 3, 4 **)** are not zero but exhibit a significant spontaneous, endogenous pro-angiogenic control activity in the OEC/pOB cultures by VEGF-A (Dohle et al., 2021). This endogenous activity is inhibited by both species of rhBMP-2 (Dohle et al., 2021), for rhBMP-2_COL_ by concentrations above 10–20 pM (see Figures 3, 4). Thus it could be argued that the observed proangiogenic activity of rhBMP-2_COL_ (Figures 2, 3) is not of a stimulatory but only of a deinhibitory nature on dilution. However such a “deinhibition” neither accounts for a 9-fold net higher angiogenic biohybrid activity *in vitro* (Table 3) nor for a severalfold net higher vessel density *in vivo* (21 days, rats) in low versus high load comparison (Al-Maawi et al., 2023) (see Tables 1, 3). The above paradox angiogenic activity agrees with the evidence of rhBMP-2_COL_ being a full inverse agonist of a novel allosteric receptor complex together with an as yet unknown orthosteric agonist (see: Berg and Clarke, 2018; de Vries et al., 2021).

## 4 Conclusion

Adsorbate load-dependent pro- and anti-angiogenic functions of the considered inverse agonist rh-BMP-2_COL_ on endothelial cells, has been determined by the novel method of solid-state biohybrids as nano- or pico-stats.

## Data Availability

The original contributions presented in the study are included in the article/supplementary material, further inquiries can be directed to the corresponding author.

## References

[B26] Al-Maawi S. Sowislok A. Dohle E. Lach R. Henning S. Michler G. H. (2023). Electrospun PDLLA nanofiber fleece loaded with low concentrated rhBMP-2 enhances bone regeneration by activating both angiogenesis and osteogenesis *in vivo* . Manuscript in preparation.

[B1] Berg K. A. Clarke W. P. (2018). Making sense of pharmacology: inverse agonism and functional selectivity. Int. J. Neuropsychopharmacol. 21, 962–977. 10.1093/ijnp/pyy071 30085126 PMC6165953

[B2] Chatzinikolaidou M. Laub M. Rumpf H. M. Jennissen H. P. (2002). Biocoating of electropolished and ultra-hydrophilic titanium and cobalt chromium molybdenium alloy surfaces with proteins. Mater. Werkst. Mat. Sci. Eng. Technol. ) 33, 720–727. 10.1002/mawe.200290002

[B3] Committee for Medicinal Products for Human use (Chmp) (2014). “InductOs-EMEA/H/C/000408-II/0100 EMA/CHMP/649027/2014,”, Assessment Report. 1–60.

[B4] Deckers M. M. van Bezooijen R. L. van der H. G. Hoogendam J. van Der B. C. Papapoulos S. E. (2002). Bone morphogenetic proteins stimulate angiogenesis through osteoblast-derived vascular endothelial growth factor A. Endocrinology 143, 1545–1553. 10.1210/endo.143.4.8719 11897714

[B27] de Vries R. M. J. M. Meijer F. A. Doveston R. G. Leijten-van de Gevel I. A. Brunsveld L. (2021). Cooperativity between the orthosteric and allosteric ligand binding sites of RORgammat. Proc.Natl.Acad.Sci U.S.A 118, 1–9. 10.1073/pnas.2021287118 PMC8017705 33536342

[B5] Dohle D. S. Zumbrink T. Meißner M. Jennissen H. P. (2020). Protein adsorption hysteresis and transient states of fibrinogen and BMP-2 as model mechanisms for proteome binding to implants. Curr. Dir. Biomed. Eng. 6, 1–4. 10.1515/cdbme-2020-3046

[B6] Dohle E. Sowislok A. Ghanaati S. Jennissen H. P. (2021). Angiogenesis by BMP-2-PDLLA-biohybrids in Co-culture with osteoblasts and endothelia. Curr. Dir. Biomed. Eng. 7, 835–838. 10.1515/cdbme-2021-2213

[B7] Food and Drug Administration (2004). INFUSE® bone Graft: Summary of safety and effectiveness data. Maryland: Access Data FDA, 1–29.

[B8] Jennissen H. P. (2019). Aspects of multimodal hybrid biomaterials. Curr. Dir. Biomed. Eng. 5, 303–305. 10.1515/cdbme-2019-0076

[B28] Jennissen H. P. (2023). Pharmaceutical composition for promoting osteoinduction and angiogenesis. Patent Application WO2023/052554A1, pp. 1–18.

[B9] Jennissen H. P. Zumbrink T. Chatzinikolaidou M. Steppuhn J. (1999). Biocoating of implants with mediator molecules: surface enhancement of metals by treatment with chromosulfuric acid. Mater. Werkst. Mat. Sci.Eng. Technol. 30, 838–845. 10.1002/(sici)1521-4052(199912)30:12<838:aid-mawe838>3.0.co;2-w

[B10] Kenley R. A. Yim K. Abrams J. Ron E. Turek T. Marden L. J. (1993). Biotechnology and bone graft substitutes. Pharm. Res. 10, 1393–1401. 10.1023/a:1018902720816 8272399

[B11] Kercheva M. Gusakova A. M. Ryabova T. R. Suslova T. E. Kzhyshkowska J. Ryabov V. V. (2020). Serum levels of bone morphogenetic proteins 2 and 4 in patients with acute myocardial infarction. Cells 9, 2179. 10.3390/cells9102179 32992577 PMC7601292

[B12] Kulikauskas M. R. Bautch V. L. (2022). The versatility and paradox of BMP signaling in endothelial cell behaviors and blood vessel function. Cell Mol. Life Sci. 79, 77. 10.1007/s00018-021-04033-z 35044529 PMC8770421

[B13] Landry J. P. Ke Y. Yu G. L. Zhu X. D. (2015). Measuring affinity constants of 1450 monoclonal antibodies to peptide targets with a microarray-based label-free assay platform. J. Immunol. Methods 417, 86–96. 10.1016/j.jim.2014.12.011 25536073 PMC4339518

[B14] Laub M. Chatzinikolaidou M. Jennissen H. P. (2007). Aspects of BMP-2 binding to receptors and collagen: influence of cell senescence on receptor binding and absence of high-affinity stoichiometric binding to collagen. Mater. Werkst. Mat. Sci. Eng. Technol.) 38, 1020–1026. 10.1002/mawe.200700238

[B15] Laub M. Seul T. Schmachtenberg E. Jennissen H. P. (2001). Molecular modelling of bone morphogenetic protein 2 (BMP-2) by 3D-rapid prototyping. Mater. Werkst. Mat. Sci. Eng. Technol. ) 32, 926–930. 10.1002/1521-4052(200112)32:12<926:aid-mawe926>3.0.co;2-1

[B16] Mayer H. Scutt A. M. Ankenbauer T. (1996). Subtle differences in the mitogenic effects of recombinant human bone morphogenetic proteins -2 to -7 on DNA synthesis on primary bone-forming cells and identification of BMP-2/4 receptor. Calcif. Tissue Int. 58, 249–255. 10.1007/bf02508644 8661956

[B17] MRI (2023). “Global bone morphogenetic protein (BMP) 2 market size & forecast 2023-2028 report,” Market research Report No:2023-2028, 1–160.

[B19] Popovic N. Wilson E. (2010). “Cell surface receptors,” in Comprehensive toxicology. Editor McQueen C. (Amsterdam: Elsevier), 81–91. 10.1016/B978-0-08-046884-6.00206-2

[B20] Sänger T. Asran A. S. Laub M. Michler G. H. Jennissen H. P. (2014). Release dynamics and biological activity of PDLLA nanofiber composites of rhBMP-2 and rhVEGF_165_ as scaffolds for tissue engineering. Biomed. Tech. Berl. 59 (S1), 69–72. 10.1515/bmt-2014-5000

[B21] Schlüter H. Apweiler R. Holzhutter H. G. Jungblut P. R. (2009). Finding one's way in proteomics: a protein species nomenclature. Chem. Cent. J. 3, 11. 10.1186/1752-153X-3-11 19740416 PMC2758878

[B22] Sowislok A. Jennissen H. P. (2022). Bioengineering of tubes, rings and panels for guided bone and vascular regeneration. Curr. Dir. Biomed. Eng. 8, 620–623. 10.1515/cdbme-2022-1158

[B23] Spanou C. E. S. Wohl A. P. Doherr S. Correns A. Sonntag N. Lutke S. (2023). Targeting of bone morphogenetic protein complexes to heparin/heparan sulfate glycosaminoglycans in bioactive conformation. FASEB J. 37, e22717. 10.1096/fj.202200904r 36563024 PMC13281839

[B24] Wiemann M. Rumpf H. M. Bingmann D. Jennissen H. P. (2001). The binding of rhBMP-2 to the receptors of viable MC3T3 cells and the question of cooperativity. Mater. Werkst. Mat. Sci. Eng. Technol. ) 32, 931–936. 10.1002/1521-4052(200112)32:12<931:aid-mawe931>3.0.co;2-h

[B25] Williams D. F. Albrektsson T. Black J. Christei P. Glantz P.-O. Gross U. (1987). “Definitions in biomaterials,” in Proceedings of a Consensus Conference of the ESB, Progress in Biomedical Engineering, Chester, England, March 3-5, 1986. Editor Williams D. F. (Elsevier Sci), pp. 66–68.

